# Corticospinal drive is associated with temporal walking adaptation in both healthy young and older adults

**DOI:** 10.3389/fnagi.2022.920475

**Published:** 2022-08-18

**Authors:** Sumire D. Sato, Julia T. Choi

**Affiliations:** ^1^Department of Applied Physiology and Kinesiology, University of Florida, Gainesville, FL, United States; ^2^Neuroscience and Behavior Program, University of Massachusetts Amherst, Amherst, MA, United States

**Keywords:** electromyography (EMG), locomotion, split-belt treadmill, gait, coherence

## Abstract

Healthy aging is associated with reduced corticospinal drive to leg muscles during walking. Older adults also exhibit slower or reduced gait adaptation compared to young adults. The objective of this study was to determine age-related changes in the contribution of corticospinal drive to ankle muscles during walking adaptation. Electromyography (EMG) from the tibialis anterior (TA), soleus (SOL), medial, and lateral gastrocnemius (MGAS, LGAS) were recorded from 20 healthy young adults and 19 healthy older adults while they adapted walking on a split-belt treadmill. We quantified EMG-EMG coherence in the beta-gamma (15–45 Hz) and alpha-band (8–15 Hz) frequencies. Young adults demonstrated higher coherence in both the beta-gamma band coherence and alpha band coherence, although effect sizes were greater in the beta-gamma frequency. The results showed that slow leg TA-TA coherence in the beta-gamma band was the strongest predictor of early adaptation in double support time. In contrast, early adaptation in step length symmetry was predicted by age group alone. These findings suggest an important role of corticospinal drive in adapting interlimb timing during walking in both young and older adults.

## Introduction

Human walking involves sequential activation of muscles during different phases of the gait cycle to control limb movement in a precise manner, and to coordinate left-right alternation between limbs. The timing and amplitude of muscle activation during walking is regulated in part by sensory feedback (Rossignol et al., 2006). Gait modifications in more challenging walking tasks (e.g., stepping over an obstacle) also requires a high degree of corticospinal input (Drew and Marigold, 2015). Specifically, the phasic drive to leg muscles from the motor cortex has been shown to increase during precision walking (Petersen et al., 2012; Jensen et al., 2018; Spedden et al., 2019). In older adults, this corticospinal drive is reduced during walking (Roeder et al., 2018; Spedden et al., 2019), which may impact one’s ability to adapt and make anticipatory adjustments to their walking pattern.

Older adults have impaired gait adaptation compared to younger adults (Bruijn et al., 2012; Nemanich and Earhart, 2015), which may lead to an increased risk of falling (Tinetti et al., 1988; Berg et al., 1997). During split-belt treadmill walking, where one leg moves faster than the other leg, healthy young adults adapt interlimb walking parameters by altering their spatial (step length) as well as temporal control (double support period) on each leg (Dietz et al., 1994; Reisman et al., 2005). Older adults can adapt interlimb parameters to the same level as young adults (Malone and Bastian, 2016; Ducharme et al., 2019; Iturralde and Torres-Oviedo, 2019; Vervoort et al., 2019a). However, the rate of adaptation is reduced in older adults greater than 70 years old (Bruijn et al., 2012; Sombric et al., 2017). The neural mechanisms that underlie age-related changes in walking control and adaptation is an active area of research (e.g., reviewed in Fettrow et al., 2021; Sato and Choi, 2021).

Electromyography coherence analysis has demonstrated a common neural drive at 15–45 Hz to the tibialis anterior that is modulated during walking adaptation (Sato and Choi, 2019; Oshima et al., 2021; Kitatani et al., 2022). During normal walking, a significant amount of coherence can be found between EMG recorded from the proximal and distal ends of the tibialis anterior in the alpha (8–15 Hz), beta (15–30 Hz), and gamma (30–45 Hz) frequencies during the swing phase of gait. Beta-gamma band EMG oscillations are thought to originate in the motor cortex and have been used as a marker of corticospinal drive (Farmer et al., 1993, 1997; Brown et al., 1998; Halliday et al., 1998, 2003). In healthy young adults, beta-band coherence in the tibialis anterior muscle is increased early during split-belt treadmill adaptation compared to baseline symmetrical walking at the slow or fast speed (Sato and Choi, 2019). Alpha-band coherence is important in slow, periodic movements (Vallbo and Wessberg, 1993), and suggested to be olivo-cerebellar in origin (Llinas and Volkind, 1973; Llinas, 2013). However, we did not observe any changes in alpha-band coherence during split-belt walking in healthy young adults (Sato and Choi, 2019), suggesting that coherence modulation during walking adaptation are specific to the beta-gamma range. Furthermore, we previously showed that beta-band intramuscular coherence was associated with double support time asymmetry but not with step length asymmetry, suggesting that corticospinal control may play a functional role in temporal control during split-belt treadmill adaptation (Sato and Choi, 2019). Therefore, we hypothesize that decreased corticospinal drive during walking older adults would have an impact on adaptation of double support time symmetry during split-belt walking.

The objective of this study was to determine the impact of aging on the contribution of corticospinal drive during split-belt locomotors adaptation. This study was a cross-sectional study between two cohorts: young (23 ± 4.6 years) and older (75 ± 4.4 years) adults. Corticospinal drive during split-belt walking adaptation was quantified by the amount of beta-gamma frequency range (15–45 Hz) coherence in the tibialis anterior (TA-TA) and plantarflexors (SOL-MGAS, MGAS-LGAS). Similar to our previous study (Sato and Choi, 2019), we also examined EMG-EMG coherence in the alpha-band (8–15 Hz) to determine if coherence modulation were frequency-specific. The overall findings were that: (1) Early change in step length asymmetry during adaptation are reduced in older adults, (2) corticospinal drive to ankle muscles is less in older adults compared to young adults, and (3) corticospinal drive to ankle muscles is associated with early changes in double support asymmetry, independent of age.

## Materials and methods

### Participants

Twenty healthy young adults and 19 healthy older adults participated in this study (Table 1). Sample size was determined based on previous studies that examined age-related differences in EMG-EMG coherence (Spedden et al., 2018, 2019; Roeder et al., 2020), and power calculation with preliminary data collected prior to this study (not published). Our desired power for age-related differences in beta-gamma coherence was 0.8 with an alpha level of 0.05. Inclusion criteria were no previous history of neurological disorder, no current orthopedic injury, ability to walk without walking aids (including ankle-foot orthoses) for at least 10 min. Participants were characterized for: physical activity using the Short Physical Performance Battery (SPPB) (Guralnik et al., 1994) and the Advanced SPPB (Simonsick et al., 2001), cognitive status using the Telephone Interview for Cognitive Status (TICS) (Brandt et al., 1988), recent subjective experience using the Fatigue Severity Scale (Krupp et al., 1988), physical activity levels using the Godin Leisure Time Questionnaire (Godin and Shephard, 1985), and leg-dominance using the Waterloo Footedness Questionnaire (Elias et al., 1998). All participants gave informed written consent before the study in accordance with the protocol approved by the Institutional Review Board of University of Florida, Gainesville, FL (Protocol # 202000764).

**TABLE 1:** Participant characteristics.

	Young (*n* = 20)	Old (*n* = 19)	*P*-value
Age (years)	23 ± 4.6	75 ± 4.4	< 0.001
Sex (M:F)	9:11	11:9	0.752
Height (cm)	169.9 ± 9.5	171.5 ± 9.0	0.581
Weight (kg)	68.2 ± 14.4	76.7 ± 16.0	0.088
BMI (kg/m^2^)	23.5 ± 3.9	26.0 ± 4.9	0.082
SPPB	11.95 ± 0.2	11.5 ± 0.9	0.049
SPPB-A	3.4 ± 0.3	3.0 ± 0.4	0.001
FSS	29.0 ± 7.8	28.1 ± 13.6	0.801
Godin	106.6 ± 112.1	191.4 ± 160.5	0.063
Waterloo	0.7 ± 0.6	0.7 ± 0.7	0.991
TICS	36.1 ± 1.7	36.2 ± 2.2	0.928

SPPB, Short Physical Performance Battery (Max score = 12; Higher score = higher physical function); SPPB-A, Advanced Short Physical Performance Battery (Max score = 4; Higher score = higher physical function); FSS, Fatigue Severity Scale (Max score = 63; Higher score = greater fatigue severity); Godin, Godin Physical Activity Questionnaire (Higher score = more physical activity); Waterloo, Waterloo Footedness Questionnaire (2 = Strong right dominance, −2 = Strong left dominance); TICS, Telephone Interview Cognitive Status (Max score = 41; Score greater than 32 = non-impaired cognitive status).

### Data collection

**FIGURE 1:**
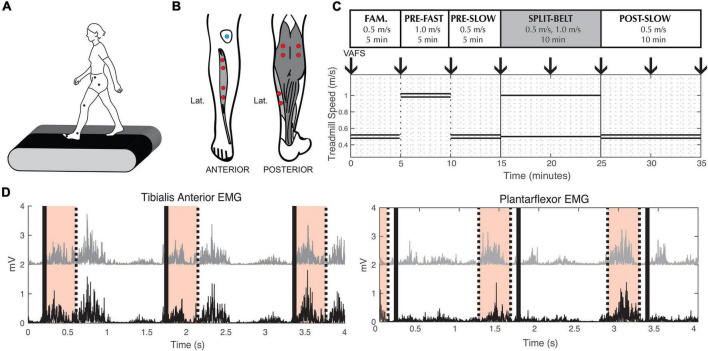
Experimental methods. **(A)** Reflective markers used to measure lower limb kinematics. **(B)** Electrode placement for Electromyography measurements. **(C)** Split-belt treadmill walking protocol. Double lines indicate treadmill speed during tied-belt conditions. Single lines indicate the different left and right speeds during split-belt condition. Down arrows indicate when participants were asked to indicate their fatigue level (VAFS). Fam. = Familiarization. **(D)** Example of processed tibialis anterior and plantarflexor EMG from a representative participant. To calculate coherence during swing phase, we used EMG signals from the proximal (black) and distal (gray) muscle belly of the tibialis anterior 0–400 ms (shaded area) after toe-off (thick black lines). To calculate coherence during stance phase, we used EMG signals from the medial gastrocnemius (black) and soleus (gray) muscle 500–100 ms (shaded area) before toe-off (thick black line).

Participants walked on an instrumented split-belt treadmill (Bertec, Columbus, OH, United States). Reflective markers were placed bilaterally on the anterior superior iliac spine (pelvis), greater trochanter (hip), joint line of the knee (knee), lateral malleolus (ankle), and 5th metatarsal (toe) (Figure 1A). Pairs of surface electrodes were placed on the muscle belly of the distal and proximal ends of the tibialis anterior (TA), medial (MGAS), and lateral (LGAS) gastrocnemius, and the soleus (SOL) on each leg (Figure 1B).

The experimental paradigm consisted of five walking conditions (Figure 1C): (1) 5 min at 0.5 m/s with tied-belt (same left and right speed) for familiarization on the treadmill, (2) 5 min at 1.0 m/s with tied-belt (“pre-fast”), (3) 5 min at 0.5 m/s with tied-belt (“pre-slow”), (4) 10 min split-belt, with one treadmill belt going at 0.5 m/s and the other at 1.0 m/s (“adaptation”), and (5) 10 min at 0.5 m/s with tied-belt (“post-slow”). The leg on the fast belt during split-belt adaptation (from here on referred to as the “fast leg”), and the leg on the slow belt (from here on referred to as the “slow leg”) was randomized between participants with the same leg dominancy (i.e., equal number of right leg dominant participants with the fast leg on the left and right sides), as leg dominancy may alter the rate of adaptation (Kong et al., 2011; Bulea et al., 2017). During the course of the experiment, subjective experience of fatigue was quantified by the Visual Analog Fatigue Scale (Figure 1C; Lee et al., 1991).

Lower limb kinematics were recorded at 100 Hz using an eight-camera Miqus system (Qualisys, Gothenburg, Sweden). Force data from the treadmill (Bertec, Columbus, OH, United States) and EMG signals from a wired amplifier (MA300, Motion Lab Systems, Baton Rouge, LA, United States) were collected at 1,000 Hz. EMG, force plate and kinematic data was synchronized using Qualisys Track Manager (Qualisys, Gothenburg, Sweden).

### Gait analysis

Ground reaction force data was low-pass filtered (3rd order Butterworth) with a 15 Hz cut-off frequency. Heel-strike and toe-off events for each leg were identified when the vertical ground reaction force crossed a threshold of 15 N (Sato and Choi, 2019). Time of heel-strike and toe-off was visually inspected and manually corrected if necessary.

Step length was calculated as the anterior-posterior distance between the ankle markers at time of heel strike. Fast and slow step lengths correspond to the leading leg being on the fast or slow belt, respectively, at heel strike (i.e., fast step = fast leg heel strike − slow limb heel-strike). Double support time was calculated as the duration when both legs were on the treadmill. Fast leg double support time correspond to the double support occurring at the beginning of the fast leg’s stance (i.e., fast leg double support = the time from fast leg heel − strike to slow leg toe-off) and the slow leg’s stance (i.e., fast leg toe-off − slow leg heel-strike), respectively. Step length asymmetry, and double support asymmetry were defined as the normalized difference between legs for each stride (Eq. 1).


(1) $$A\,s\,y\,m\,m\,e\,t\,r\,y=\frac{F\,a\,s\,t\,l\,e\,g-S\,l\,o\,w\,l\,e\,g}{F\,a\,s\,t\,l\,e\,g+S\,l\,o\,w\,l\,e\,g}$$

Averaged values were calculated over three different epochs during adaptation and post-adaptation: (1) Initial (mean of first five strides), (2) Early adaptation/post-adaptation (mean of strides #6–30), and (3) plateau (mean of last 30 strides) (Leech and Roemmich, 2018; Leech et al., 2018). Baseline asymmetry was calculated from the first five strides of pre-slow and pre-fast. Overall change in adaptation and post-adaptation was identified as the asymmetry difference between plateau and initial epochs during split-belt adaptation and post-adaptation, respectively. Similarly, early change was identified as the asymmetry difference between early and initial epochs during split-belt adaptation and post-adaptation.

### Coherence analysis

Coherence between EMG pairs (denoted *x* and *y*) was characterized based on previously described methods and MATLAB functions from NeuroSpec.^1^ EMG signals were high-pass filtered at 8 Hz, rectified, and normalized to have unit variance (Halliday et al., 1995). Discrete Fourier transformation analysis was applied to short sections of the EMG taken at a fixed offset time to estimate their average autospectras, *f*_*xx*_ and *f*_*yy*_, and cross-spectrum *f*_*xy*_. Based on preliminary data, we used 0–400 ms after toe-off to calculate TA-TA coherence, and 500–100 ms before toe-off to calculate SOL-MGAS (plantarflexor) coherence and MGAS-LGAS (gastrocnemius) coherence (Figure 1D). For each Fourier frequency (λ), the resulting coherence value provides a measure of association of the *x* and *y* processes on a scale from 0 to 1 (Eq. 2). A coherence value of 0 signifies no synchrony between the two EMG signals and a coherence value of 1 signifies perfect synchrony between the two EMG signals.


(2) $${\left|R_{x\,y}\,\left(\lambda \right)\right|}^{2}=\frac{{\left|f_{x\,y}\,\left(\lambda \right)\right|}^{2}}{f_{x\,x}\,\left(\lambda \right)\,f_{y\,y}\,\left(\lambda \right)}$$

To characterize coherence modulation over the course of locomotor adaptation, coherence was calculated over the first 100 strides during each baseline condition (pre-slow, pre-fast), and over the first and last 100 strides during split-belt adaptation (early and late adaptation) and post-adaptation (early and late post-adaptation) period.

The natural logarithm of the cumulative sum of coherence was calculated for the beta-gamma band (15–45 Hz) to quantify corticospinal drive to the lower limb muscles for each condition. Since EMG-EMG coherence in the alpha-band is thought to originate from a different central nervous system source compared to the beta-gamma band (although there are some studies that challenge this view; Salenius et al., 1997; Mima and Hallett, 1999; Graziadio et al., 2010), we also calculated coherence in the alpha band (8–15 Hz) to examine if alpha band modulation is different from beta-gamma band modulation. All together, there were a total of 12 coherence measures (2 legs × 3 EMG pairs × 2 frequency bands) for each condition.

### Statistical analysis

Age group differences in overall and early changes in kinematic adaptation were assessed though independent *t*-tests. Effect sizes for paired comparisons were calculated with Cohen’s d; defined as small < 0.499, moderate = 0.500–0.799 and large > 0.800. Since group characteristics demonstrated that physical function was different between groups (Table 1, for Advanced SPPB and SPPB), we used an analysis of covariate to examine group differences in kinematic changes controlling for physical function.

Two-way mixed measures ANOVA was performed to determine the effects of Age (Young vs. Older) and Condition (pre-fast, pre-slow, early adaptation, late adaptation, early post-adaptation, and late post-adaptation) on each coherence measure. Greenhouse-Geisser corrections were applied when the assumption of sphericity was violated (Mauchly’s test: *p* = 0.05) and epsilon was less than 0.75. Huynh-Feldt corrections were applied when the assumption of sphericity was violated (Mauchly’s test: *p* = 0.05) and epsilon was greater than 0.75. *Post hoc* pairwise comparisons were conducted with Bonferroni corrections. Effect sizes for ANOVAs were determined by partial eta-squared (η^2^_*p*_); defined as small < 0.059, moderate = 0.060–0.139, and large > 0.140.

Forward stepwise regression was used to determine which coherence measures best predicted individual differences in kinematic adaptation (early change in step length and double support). Age group and six beta-gamma coherence measures from early adaptation were included as co-variates.

All statistical significance was established with an alpha level = 0.05. Statistical analyses were performed using JASP v0.14.1 (University of Amsterdam, Amsterdam, Netherlands).

## Results

### Aging influences kinematic changes during split-belt locomotor adaptation

**FIGURE 2:**
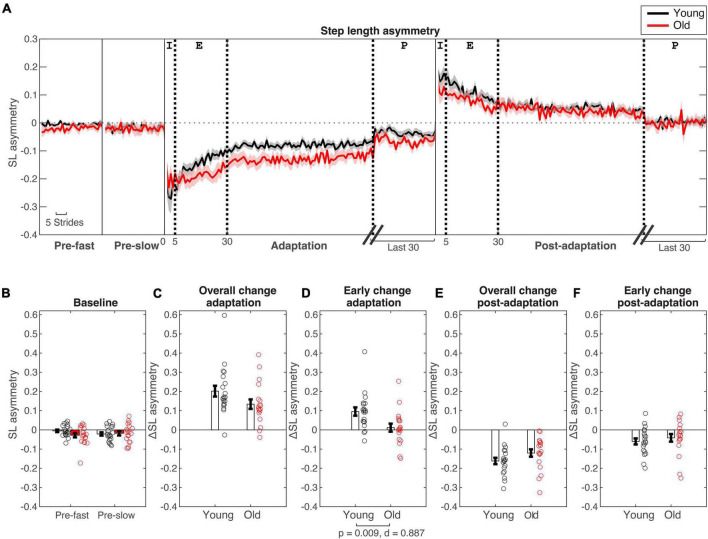
Step length asymmetry changes during split-belt adaptation. **(A)** Stride-by stride changes in step length asymmetry plotted for young (in black) and older adults (in red). Shaded areas are standard errors. For baseline (“pre-”) conditions the first 30 strides are plotted. For adaptation and post-adaptation conditions, the first 100 and last 30 strides are plotted. Thick dotted lines are for stride numbers 5, 30, and 100/30 strides before the last stride to indicate the different epochs [Epochs indicated at top of A: Initial (I) = Strides #1–5, early (E) = Strides #6–30, plateau (P) = Last 30 strides]. **(B–F)** Age group means and standard error bars for **(B)** baseline-fast and pre-slow conditions, **(C)** Overall change during adaptation (Δ plateau–initial), **(D)** early change during adaptation (Δ earlyinitial), **(E)** Overall change during post-adaptation (Δ plateau–initial), **(F)** early change during post-adaptation (Δ early–initial).

**FIGURE 3:**
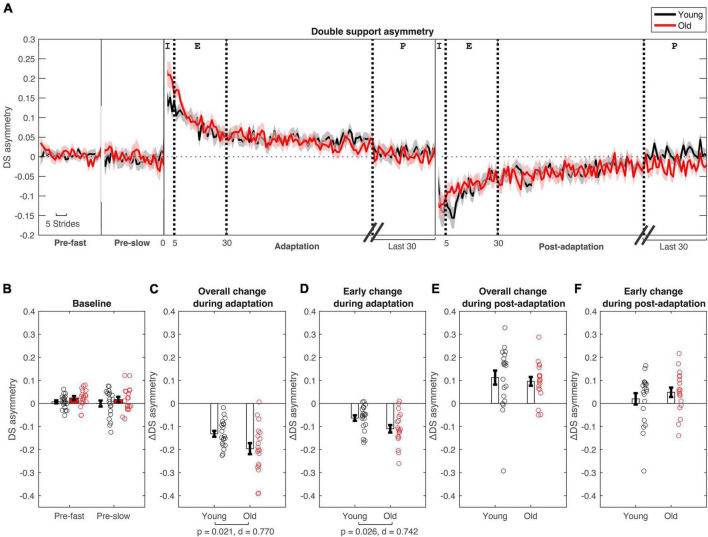
Double support asymmetry changes during split-belt adaptation. **(A)** Stride-by stride changes in double support asymmetry plotted for young (in black) and older adults (in red). **(B–F)** Age group means and standard error bars for **(B)** baseline-fast and pre-slow conditions, **(C)** Overall change during adaptation (Δ plateau–initial), **(D)** early change during adaptation (Δ early–initial), **(E)** Overall change during post-adaptation (Δ plateau–initial), **(F)** early change during post-adaptation (Δ early–initial). See Figure 2 caption for description of epochs.

Participants walked with symmetrical spatial and temporal kinematics during pre-slow and pre-fast; there was no evidence of age group differences during baseline conditions for any kinematic asymmetry variables (Table 2 and Figures 2B, 3B). In general, VAFS showed an increase in fatigue during the protocol in both young and older adults, but there were no group differences [*F*(1,36) = 0.44, *p* = 0.511, η^2^_*p*_ = 0.012].

**TABLE 2:** Age group differences in baseline kinematic asymmetry.

Condition	Asymmetry variables	*P*-value	95% Confidence interval for		Effect size
			difference in group means		
			Lower	Upper	
Pre-fast	Step length	0.078	−0.003	0.05	0.580
	Double support	0.121	−0.04	0.01	−0.508
Pre-slow	Step length	0.806	−0.03	0.03	−0.079
	Double support	0.342	−0.05	0.02	−0.308

Group differences are analyzed with a student t-test, and effect size is given by Cohen’s d.

During initial split-belt treadmill adaptation, participants had longer step lengths on the slow leg compared to the fast leg, leading to negative asymmetry (Figure 2A). Participants gradually adapted and reached a plateau. During post-adaptation, there was an after-effect in which participants took longer steps on the fast leg and gradually de-adapted to reach a plateau. Overall change in step length asymmetry (Δ plateau phase−initial phase) during adaptation was not different between age groups (*p* = 0.079; Figure 2C and Table 3), but early change in step length asymmetry was greater in younger adults compared to older adults (*p* = 0.009; Figure 2D). Overall and early change in step length asymmetry during post-adaptation was not significantly different between age groups (Overall Δ: *p* = 0.111; Early Δ: *p* = 0.464; Figures 2E,F). After controlling for physical function, overall change in step length asymmetry during adaptation was not different between groups, but there was a significant effect of age groups in early change in step length asymmetry even when adjusting for SPPB-A scores [Overall Δ: *F*(1, 36) = 2.20, *p* = 0.146, *n*^2^_*p*_ = 0.058; Early Δ: *F*(1, 36) = 9.30, *p* = 0.004, *n*^2^_*p*_ = 0.205], which was consistent with the results above. Comparisons for post-adaptation step length asymmetry changes were consistent with reported above and were not statistically significant after adjusting for SPPB-A scores (all *p*’s > 0.100).

**TABLE 3:** Age group differences in kinematic asymmetry during adaptation and post-adaptation.

Condition	Asymmetry variables	Difference	*P*-value	95% Confidence interval for		Effect size
				difference in group means		
				Lower	Upper	
Adaptation	Step length	Overall change	0.079	−0.01	0.14	0.578
		Early change	0.009	0.02	0.15	0.887
	Double support	Overall change	0.021	0.01	0.12	0.770
		Early change	0.026	0.01	0.09	0.742
Post-adaptation	Step length	Overall change	0.111	−0.09	0.01	−0.523
		Early change	0.464	−0.07	0.03	−0.237
	Double support	Overall change	0.657	−0.06	0.09	0.143
		Early change	0.382	−0.09	0.04	−0.283

Group differences were analyzed with a student t-test, and effect size is given by Cohen’s d.

Participants had longer double support time on the fast leg compared to the slow leg, during initial split-belt treadmill adaptation, leading to positive asymmetry. Participants gradually adapted and reached a plateau close to symmetry. During post-adaptation, there was an after-effect in which participants took longer double support time on the slow leg and gradually de-adapted to reach a plateau (Figure 3A). Overall and early change in double support time asymmetry during adaptation was different between age groups (Overall Δ: *p* = 0.021; Early Δ: *p* = 0.026). Older adults adapted more overall, and demonstrated greater early change (i.e., more negative) in double support asymmetry during split-belt treadmill adaptation compared to younger adults (Figures 3C,D). During post-adaptation, overall and early change in double support asymmetry was not significantly different between age groups (Overall Δ: *p* = 0.657; Early Δ: *p* = 0.382; Figures 3E,F). When controlled for physical function, overall and early change in double support asymmetry during adaptation was not different between groups when adjusting for SPPB-A scores [Overall Δ: *F*(1, 36) = 1.68, *p* = 0.203, *n*^2^_*p*_ = 0.045; Early Δ: *F*(1, 36) = 3.00, *p* = 0.092, *n*^2^_*p*_ = 0.077]. Comparisons for post-adaptation double support asymmetry changes were consistent with reported above and were not statistically significant after adjusting for SPPB-A scores (all *p*’s > 0.100).

### Electromyography-electromyography coherence differences between age-groups

**FIGURE 4:**
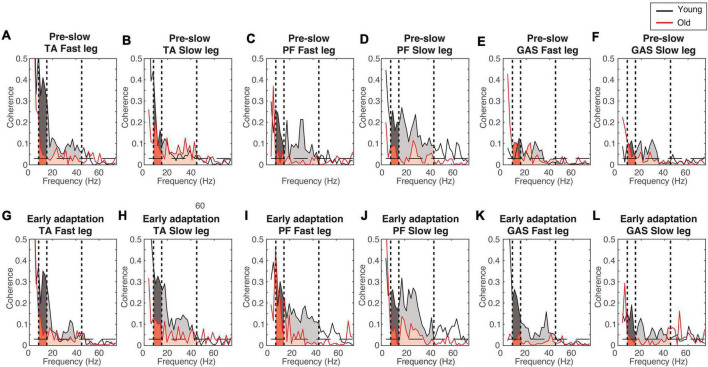
Example coherence from a representative young and old participant. **(A–F)** Pre-slow coherence in the tibialis anterior **(A,B)**, plantarflexors **(C,D)**, and gastrocnemius **(E,F)**. **(G–L)** Early adaptation coherence in the tibialis anterior **(G,H)**, plantarflexors **(I,J)**, and gastrocnemius **(K,L)**. Dashed horizontal lines indicate the 95% confidence limit. Black = Young; Red = Old; Darker shaded areas = alpha-band frequency (8–15 Hz); Lighter shaded areas = beta-gamma band frequency (15–45 Hz).

**FIGURE 5:**
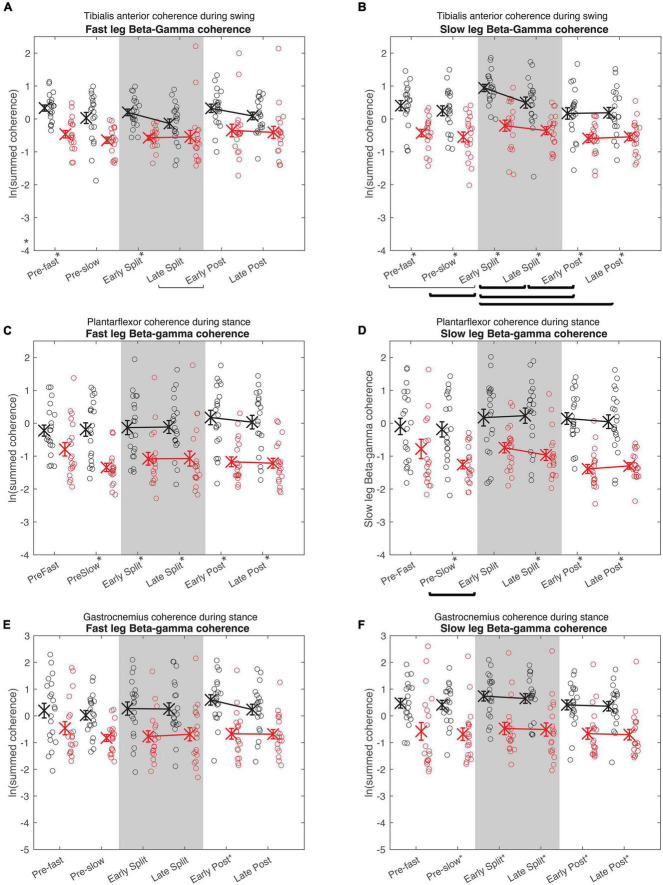
Cumulative beta-gamma electromyography-electromyography coherence. Intramuscular coherence between the distal and proximal tibialis anterior during swing phase **(A,B)**, intermuscular coherence between the medial gastrocnemius and soleus during stance phase **(B,C)**, and coherence between the medial and lateral gastrocnemius during stance phase **(E,F)** in the fast **(A,C,E)** and slow leg **(B,D,F)**. Black = Young; Red = Old; X = group means; Error bars = standard error. Brackets indicate between-condition comparisons where *p* < 0.05. Thick brackets indicate speed-matched between-condition comparisons where *p* < 0.05. * indicates between-groups comparisons within condition where *p* < 0.05. All comparisons were corrected for multiple comparisons using the Bonferroni method.

**FIGURE 6:**
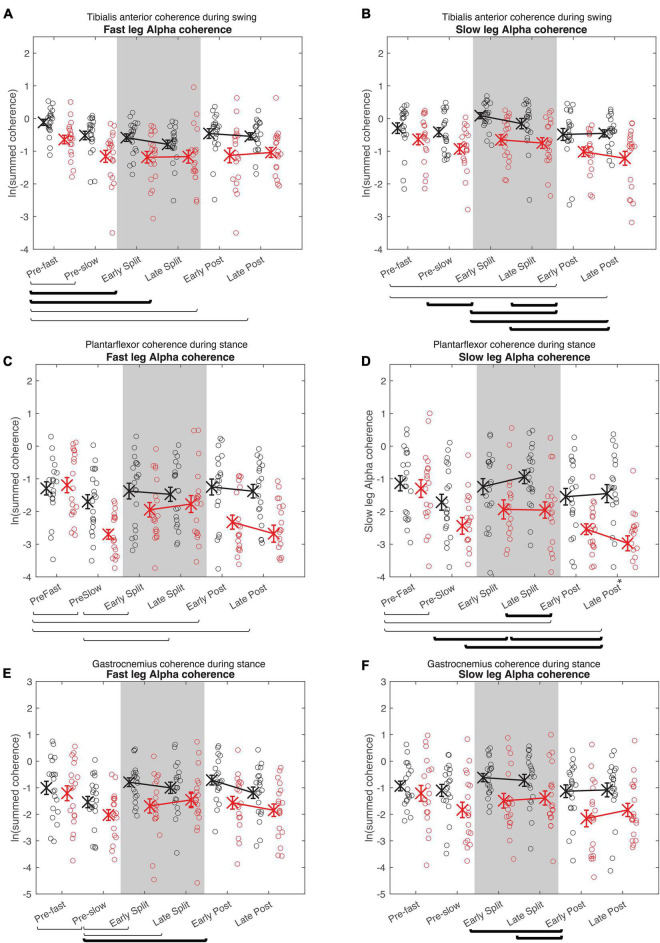
Cumulative alpha electromyography-electromyography coherence. Intramuscular coherence between the distal and proximal tibialis anterior during swing phase **(A,B)**, intermuscular coherence between the medial gastrocnemius and soleus during stance phase **(B,C)**, and coherence between the medial and lateral gastrocnemius during stance phase **(E,F)** in the fast **(A,C,E)** and slow leg **(B,D,F)**. See Figure 5 caption for description of symbols.

Figure 4 shows the EMG-EMG coherence from a representative young and old participant. Mixed-measures ANOVA statistics are summarized in Tables 4, 5. All coherence had significant main effect of age groups. All coherence except fast leg plantarflexor beta-gamma coherence and gastrocnemius beta-gamma coherence had a significant main effect of conditions. Since treadmill speed may influence coherence, only speed-equivalent comparisons (i.e., Fast leg: pre-fast vs. split-belt, pre-slow vs. post-slow, Slow leg: pre-slow vs. split-belt, and pre-slow vs. post-slow) are reported in the text and highlighted in bold brackets in Figures 5, 6.

**TABLE 4:** Main effect of condition and group × condition interaction for coherence.

			Main effect of condition				Interaction effect				Residuals
			df	*F*	*p*	η^2^_*p*_	df	*F*	*p*	η^2^_*p*_	df
Beta-gamma	Tibialis anterior	Fast leg*	2.49	3.24	0.034	0.081	2.49	1.13	0.336	0.030	92.10
		Slow leg^+^	4.35	11.86	< 0.001	0.243	4.35	1.42	0.227	0.037	160.96
	Plantarflexors	Fast leg*	3.07	1.19	0.317	0.031	3.07	2.48	0.063	0.063	113.60
		Slow leg*	2.47	3.00	0.044	0.075	2.47	2.37	0.088	0.060	91.22
	Gastrocnemius	Fast leg*	2.88	1.70	0.173	0.044	2.88	1.17	0.323	0.031	106.70
		Slow leg*	2.62	1.89	0.143	0.049	2.62	0.12	0.929	0.003	97.06
Alpha	Tibialis anterior	Fast leg	5.00	7.60	< 0.001	0.170	5.00	0.52	0.762	0.014	185.00
		Slow leg^+^	4.27	9.99	< 0.001	0.213	4.27	1.37	0.246	0.036	157.80
	Plantarflexors	Fast leg^+^	4.32	7.91	< 0.001	0.176	4.32	4.95	< 0.001	0.118	159.94
		Slow leg*	2.81	9.98	< 0.001	0.212	2.81	3.12	0.032	0.078	103.94
	Gastrocnemius	Fast leg*	3.09	4.55	0.004	0.110	3.09	1.13	0.339	0.030	114.031
		Slow leg*	2.73	4.47	0.007	0.108	2.73	1.03	0.337	0.027	101.14

* = Greenhouse-Geisser correction was applied;

^+^ = Huynh-Feldt correction was applied.

**TABLE 5:** Main effect of age groups for coherence.

			Main effect of group					Residuals
			df	*F*	*p*	η^2^_*p*_	Cohen’s d	df
Beta-gamma	Tibialis anterior	Fast leg	1	19.29	< 0.001	0.343	0.703	37
		Slow leg	1	25.52	< 0.001	0.408	0.809	37
	Plantarflexors	Fast leg	1	22.82	< 0.001	0.381	0.765	37
		Slow leg	1	24.82	< 0.001	0.401	0.798	37
	Gastrocnemius	Fast leg	1	14.29	< 0.001	0.279	0.605	37
		Slow leg	1	19.39	< 0.001	0.344	0.705	37
Alpha	Tibialis anterior	Fast leg	1	11.05	0.002	0.230	0.532	37
		Slow leg	1	9.96	0.003	0.212	0.505	37
	Plantarflexors	Fast leg	1	9.10	0.005	0.197	0.483	37
		Slow leg	1	10.88	0.002	0.227	0.528	37
	Gastrocnemius	Fast leg	1	6.55	0.015	0.150	0.410	37
		Slow leg	1	7.00	0.012	0.159	0.424	37

In both the fast and slow leg, beta-gamma TA-TA coherence swing phase was different between conditions and between groups, but condition × group interaction was not significant (Figure 5A Fast leg; Condition: *p* = 0.034; Group: *p* < 0.001; Interaction: *p* = 0.336) (Figure 5B Slow leg; Condition: *p* < 0.001; Group: *p* < 0.001; Interaction: *p* = 0.227). Beta-gamma TA-TA coherence during swing phase was lower in older adults compared to younger adults. In the fast leg, speed-equivalent conditions did not show any statistically significant differences. In the slow leg, early split-belt coherence was higher compared to baseline pre-slow, late split-belt, and early and late post-adaptation, and late split-belt adaptation was higher compared to early post-adaptation.

For plantarflexor (SOL-MGAS) coherence, beta-gamma coherence in the fast leg during stance phase was different between groups, but not between conditions, and condition × group effect was not significant (Figure 5C; Condition: *p* = 0.317; Group: *p* < 0.001; Interaction *p* = 0.063). While in the slow leg, beta-gamma plantarflexor coherence during stance phase was different between conditions and between groups, and condition × group effect was not significant (Figure 5D; Condition: *p* = 0.044; Group: *p* < 0.001; Interaction: *p* = 0.088). Beta-gamma plantarflexor coherence was lower in older adults compared to younger adults in both legs. *Post-hoc* between condition tests for the slow leg showed that coherence during early split-belt was higher compared to baseline pre-slow.

In both legs, beta-gamma-band gastrocnemius (MGAS-LGAS) coherence in the fast leg during stance phase was different between groups, but not between conditions, and condition × group effect was not significant (Figure 5E; Fast leg; Condition: *p* = 0.173; Group: *p* < 0.001; Interaction: *p* = 0.323) (Figure 5F; Slow leg; Condition: *p* = 0.143; Group: *p* < 0.001; Interaction: *p* = 0.929). Beta-gamma-band gastrocnemius coherence in both legs was lower in older adults compared to younger adults.

For all alpha band coherence, there was a significant difference between conditions and between groups (younger adults > older adults), but condition × group effect was not significant (Figure 6 and Tables 4, 5). *Post-hoc* tests between conditions showed that pre-fast alpha-band coherence in the tibialis anterior in the fast leg was higher compared to all the other conditions. In the slow leg, TA-TA alpha band coherence was higher during early split-belt compared to baseline-pre-slow and early and late post-adaptation. Slow leg TA-TA alpha coherence during late split-belt coherence was not significantly higher compared to pre-slow, but was higher compared to early and late post-adaptation.

*Post-hoc* between condition tests showed that baseline pre-fast plantarflexor alpha-band coherence during pre-fast was higher compared to pre-slow, and early and late post-adaptation. Fast leg plantarflexor alpha-band coherence during pre-slow was lower compared to early and late post-adaptation. In the slow leg, *post-hoc* between condition tests showed that plantarflexor alpha-band coherence increased during split-belt adaptation in which during early adaptation coherence was higher compared to late post-adaptation, and late adaptation coherence was higher compared to pre-slow and early and late post-adaptation conditions.

For fast leg gastrocnemius alpha band coherence, *post-hoc* between conditions tests showed that coherence during pre-slow was lower compared to early post-adaptation. In the slow leg, gastrocnemius alpha band coherence during early and late adaptation was higher compared to early post-adaptation.

### Coherence predicts early changes in double support time adaptation

Early change in step length asymmetry was predicted by Age group alone [β = −0.084, *F*(1, 37) = 7.70, *p* = 0.009]. None of the coherence values during early adaptation contributed to the model for early changes in step length asymmetry during adaptation (Table 6). For early change in double support asymmetry during adaptation, > 30% of the variance can be accounted for by two variables [*r*^2^ = 0.303, *F*(2,36) = 7.82, *p* = 0.002]: slow leg tibialis anterior beta-gamma coherence was the strongest predictor (Slow leg TA: β = 0.046, *p* < 0.001), followed by fast leg plantarflexor coherence (Fast leg PF: β = −0.028, *p* = 0.009).

**TABLE 6:** Multiple linear regression models.

Early change in SL adaptation (Δ Early − Initial)					
Model: *F*(1, 37) = 7.70, *p* = 0.009; *r*^2^ = 0.172, RMSE = 0.094					

Coefficients entered in model	Unstandardized β	Standard error	**p**	95% confidence interval	
				Upper	Lower
Intercept	0.179	0.047	< 0.001	0.083	0.276
Group	−0.084	0.03	0.009	−0.145	−0.022

**Early change in DS adaptation (Δ Early − Initial)**					
**Model: *F*(2,36) = 7.82, *p* = 0.002; *r*^2^ = 0.303, RMSE = 0.057**					

Intercept	−0.120	0.013	< 0.001	−0.147	−0.094
Slow leg TA beta-gamma band coherence	0.046	0.012	< 0.001	0.021	0.071
Fast leg PF beta-gamma band coherence	−0.028	0.01	0.009	−0.049	−0.008

RMSE, root mean square error; SL, step length; DS, double support; TA, tibialis anterior; PF, plantarflexors.

## Discussion

### Aging influences both spatial and temporal control during split-belt locomotor adaptation

Older adults adapted the same amount of step length asymmetry, but early changes during adaptation were less compared to younger adults. In addition, the effect size in overall change in step length asymmetry was moderate (*d* = 0.578), which may suggest that older adults have a slightly smaller overall change in step length asymmetry compared to younger adults. The smaller early change in step length asymmetry in older adults agrees with previous split-belt studies that evaluated similar age-groups and showed a slower rate of adaptation (Bruijn et al., 2012; Sombric et al., 2017).

Both overall and early changes in double support asymmetry adaptation were different between older and younger adults. Our results on overall adaptation on double support asymmetry are in contrast with previous studies on aging (Vervoort et al., 2019a,b). This difference in outcome may reflect a variety of factors that can contribute to aging that impact the study population (e.g., level of daily physical activity, cognitive function, etc.) but it is important to note that our participant group was older compared to the other studies (Vervoort et al., 2019a older adults age: 55.3 ± 2.9 years; Vervoort et al., 2019b older adults age: 67.8 ± 5.8 years). Here we found a larger early change in double support asymmetry during adaptation in older adults compared to younger adults. However, when adjusted for physical function (Advanced SPPB scores), the age-group differences in double support asymmetry were not statistically significant. This may suggest that the strategy to alter double support asymmetry is a compensatory strategy in older adults with decreased physical function.

### Aging influences corticospinal drive during split-belt locomotor adaptation

Corticospinal drive quantified by beta-gamma coherence was lower in older adults compared to younger adults. This is in agreement with previous studies that examined EMG-EMG coherence in older and younger adults during walking (Spedden et al., 2019; Dos Santos et al., 2020; Gennaro and de Bruin, 2020). This finding is interesting and important because multiple studies have reported increased demand in cortical brain resources, especially during walking in older adults compared to younger adults (Chen et al., 2017; Mirelman et al., 2017; Hawkins et al., 2018). This may suggest that even though older adults may engage more cortical resources during walking compared to younger adults, the output from the motor cortex that reaches the muscle is reduced, which may be due to the physiological changes in the corticospinal structures [e.g., decrease in the number of motor units, decrease in the innervation ratio of muscle fiber:motor neuron (Deschenes, 2011)].

In general, older adults also demonstrated less modulation in corticospinal drive compared to younger adults. The increase in slow leg beta-gamma TA-TA coherence from baseline pre-slow to early split-belt adaptation observed in younger adults is less in older adults. This is in agreement with a previous study that also demonstrated a reduced modulation in plantarflexor intramuscular coherence during different standing balance tasks in older adults compared to younger adults (Watanabe et al., 2018). Increase in age has been associated with altered transcranial magnetic stimulation output that signify corticospinal excitability (Rossini et al., 2007), and intra-(Peinemann et al., 2001; Fujiyama et al., 2012) and inter-cortical inhibition (Talelli et al., 2008; Fling and Seidler, 2012), which may affect the ability for older adults to modulate corticospinal drive during walking adaptation. Fatigue can also modulate beta-band EMG-EMG coherence (Dos Santos et al., 2020), which may have influenced the changes in coherence during this study. However, we did not observe consistent beta-gamma coherence differences between pre-slow and post-slow that would be explained by fatigue. Alternatively, reduced modulation in beta-gamma coherence during split-belt adaptation may reflect less cortical involvement, due to greater reliance on implicit processes (Kitatani et al., 2022).

Older adults also demonstrated lower alpha-band coherence compared to younger adults, but effect size was generally smaller compared to beta-gamma coherence group differences. In contrast to beta-gamma coherence, where older adults showed less modulation in coherence compared to younger adults, older adults modulated alpha-band coherence more compared to younger adults (as indicated by the significant interaction effect in the alpha band coherence in the plantarflexors). Corticomuscular coherence in the alpha band has been suggested to be related to processing of sensory feedback (Hansen and Nielsen, 2004), and error processing (Mehrkanoon et al., 2014) which is in line with the cerebellum’s importance with sensorimotor processing (For review: Manto et al., 2012; Baumann et al., 2015; Sokolov et al., 2017).

### Corticospinal drive is associated with temporal adaptation

None of the coherence measures during early adaptation predicted step length adaptation. Only the age grouping variable significantly contributed to predict early change in step length asymmetry adaptation. Statistically, this is equivalent to an independent *t*-test and is consistent with the group comparisons. This suggests that corticospinal drive to the major lower limb ankle muscles is not associated with the early changes in step length asymmetry. Previous studies have demonstrated intact step length adaptation during split-belt walking even after cerebral lesions (Choi et al., 2009; Reisman et al., 2013). Together, this suggests that reduced corticospinal drive could be compensated by other neural mechanisms to adapt step length symmetry.

Slow leg tibialis anterior beta-gamma coherence and fast leg plantarflexor beta-gamma coherence significantly contributed to predict early change in double support asymmetry. Larger beta-gamma coherence in the slow leg tibialis anterior and smaller beta-gamma coherence in the fast leg plantarflexors was related to smaller (i.e., less negative) early change in double support asymmetry during adaptation. Higher intermuscular coherence in the beta-gamma band has been shown to be indicative of functional coordination (i.e., synergy), while lower intermuscular coherence has been indicative of greater individual muscle control (Laine and Valero-Cuevas, 2017). Therefore, in addition to the corticospinal drive to the tibialis anterior, synergy in the plantarflexor may be important for changes in double support asymmetry during split-belt adaptation. This suggests that more corticospinal drive is not necessarily always “better”; to make appropriate gait adjustments, but there should be a balance of corticospinal drive to each muscle or muscle groups which is specific to the desired outcome.

## Conclusion

During split-belt locomotor adaptation, corticospinal drive was less in older adults compared to younger adults. In both age groups, the corticospinal drive to the tibialis anterior during the swing phase was the strongest predictor of early temporal changes. This suggests that corticospinal drive plays an important role in locomotion adaptation, and that age-related changes in corticospinal drive may necessitate different locomotor adaptation strategies with increased age.

## Data availability statement

The data that support the findings of this study will be made available upon reasonable request to the corresponding author.

## Ethics statement

The studies involving human participants were reviewed and approved by University of Florida. The participants provided their written informed consent to participate in this study.

## Author contributions

SS collected, processed and analyzed the data, prepared the figures and tables, and wrote the first draft of the manuscript. Both authors designed the experiment, interpreted the results, revised, and approved of the final manuscript.

## Footnotes

1
http://www.neurospec.org
